# Antioxidant, Iron Chelating and Tyrosinase Inhibitory Activities of Extracts from *Talinum triangulare* Leach Stem

**DOI:** 10.3390/antiox2030090

**Published:** 2013-07-17

**Authors:** Ana Paula Oliveira Amorim, Márcia Cristina Campos de Oliveira, Thiago de Azevedo Amorim, Aurea Echevarria

**Affiliations:** 1 Department of Chemistry, Institute of Exact Sciences, Universidade Federal Rural do Rio de Janeiro, Seropédica, Rio de Janeiro 23890-000, Brazil; E-Mails: amorim@ufrrj.br (A.P.O.A.); echevarria@ufrrj.br (A.E.); 2 Department of Botanic, Institute of Biology, Universidade Federal Rural do Rio de Janeiro, Seropédica, Rio de Janeiro 23890-000, Brazil; E-Mail: thiagodb@ufrrj.br

**Keywords:** portulacaceae, DPPH, Fe II ions, oxidase, tyrosinase

## Abstract

The aim of this work is to evaluate the antioxidant activity against the radical species DPPH, the reducing capacity against Fe II ions, and the inhibitory activity on the tyrosinase enzyme of the *T. triangulare*. Hydromethanolic crude extract provided two fractions after the liquid/liquid partition with chloroform. The Folin-Ciocalteu method determined the total phenolic content of the crude extract (CE) and the hydromethanolic fraction (Fraction 1), resulting in a concentration of 0.5853 g/100 g for Fraction 1, and 0.1400 g/100 g for the CE. Taking into account the results of the DPPH, the free radical scavenging capacity was confirmed. The formation of complexes with Fe II ions was evaluated by UV/visible spectrometry; results showed that CE has complexing power similar to the positive control (*Gingko biloba* extract).The inhibitory capacity of samples against the tyrosinase enzyme was determined by the oxidation of L-DOPA, providing IC_50_ values of 13.3 μg·mL^−1^ (CE) and 6.6 μg·mL^−1^ (Fraction 1). The values indicate that Fraction 1 was more active and showed a higher inhibitory power on the tyrosinase enzyme than the ascorbic acid, used as positive control. The hydromethanolic extract of *T. triangulare* proved to have powerful antioxidant activity and to inhibit the tyrosinase enzyme; its potential is increased after the partition with chloroform.

## 1. Introduction

Plant invaders adversely affect key components of biological diversity, including species diversity [1,2] and ecosystem processes [3]. In addition, invasive species have a considerable socioeconomic impact. Its effect can be seen in most ecosystems; invasion represents a major conservation challenge for land management agencies [4]. However, many invasive plants are also used as food and medicine.

The Portulacaceae is a relatively small family (30 genera and 400 species) of the Caryophyllales order, but it has widespread distribution. Portulacaceae species are generally small herbaceous plants. Most of the family members have fleshy to fully succulent leaves. They live in diverse habitats, and some of them, such as *Portulaca oleracea*, *Portulaca pilosa* and *Talinum triangulare*, are considered invasive [5].

The *T. triangulare* species, which is known as “cariru” in Brazil, is a non-conventional vegetable crop of the Portulacaceae family, is mainly consumed in Northern Brazil, especially in the states of Pará and Amazonas, where the soft and highly nutritious leaves are used as a substitute for spinach. It is well adapted to the hot and humid climate and the poor quality soil, which makes its cultivation an important economic activity for small growers. This species is used in traditional medicine as a tonic and enhance the cognitive ability; however, few studies have investigated its medicinal effects [6,7].

Early studies proved that hydromethanolic extract from the branches of *T. triangulare* is a source of allantoin, aspartic acid and steroidal saponin mixture, besides the extract showed inhibitory effect against *Tripanossoma cruzi* [8,9]. Recently, HPLC analysis has shown the presence of flavonoids in *T. triangulare* extract [10], but there has not been any report on the isolation or structural characterization of the metabolites class.

It is known that oxygen free-radicals, the reactive oxygen species (ROS), are involved in cancer, diabetes, cardiovascular diseases, neurodegenerative diseases, ageing [11,12] and various acute and chronic liver diseases [13]; therefore, it is believed that antioxidant agents are helpful for treatments [14]. An increasing number of studies have indicated that many polysaccharides extracted from plants possess potent antioxidant abilities [15]. Recent studies have found that polysaccharides isolated from *T. triangulare* water extract show remarkably different degrees of antioxidant activities in dose-dependent manners [16].

Due to the ethnobotanical and feed importance of the *T. triangulare* and the absence of studies that prove their antioxidant activity, in this paper, we report the metabolite classes screening from *T. triangulare* stems which have been extracted with methanol/water. Was determined the total phenolic content and evaluated the antioxidant using DPPH radical scavenging activity and iron chelating, and tyrosinase inhibition properties.

## 2. Experimental Section

### 2.1. Plant

*Talinum triangulare* Leach sample was collected in Guapimirim, Rio de Janeiro, Brazil. This species was identified by botanist Pedro Germano Filho, and a voucher specimen (SBR26906) is deposited at the Herbário RBR of Universidade Federal Rural do Rio de Janeiro, Department of Botany. Stem of the specie under study was dried in an oven, at an average temperature of 40 °C, for some days. The dried steam (403 g) was macerated with methanol:water (8:2) at room temperature, with solvent changes every 48 h. The hydroalcoolic solution was concentrated under reduced pressure, at 60 °C, affording the crude extract (CE). After, the crude extract was partitioned with CHCl_3_ to produce a methanol/water fraction (Fraction 1) and a chloroform fraction (Fraction 2).

To investigate the special metabolite classes, a chemical screening of the sample fractions was performed using the method described by Matos [17].

*Ginkgo biloba* powdered purchased from Herbarium company was extracted with methanol:water (9:1) by 48 h at room temperature. The solution was concentrated under reduced pressure, at 60 °C, after the crude extract was used for assay of the iron binding.

### 2.2. Determination of Total Phenolics

The total phenolic content of the crude extract and Fraction 1 were determined colorimetrically using the Folin-Ciocalteau method as described by Silva [18]. To achieve this purpose, 2.0 mL (1.0 mg/mL in H_2_O milli-Q) aliquots of the fractions were added to 2.5 mL of recently prepared Folin-Ciocalteau reagent (Sigma-Aldrich), followed by the addition of 5 mL of an aqueous 14% sodium carbonate solution. The mixture was stirred and allowed to stand for 2 h. The absorbance at 760 nm was measured using a UV/VIS spectrophotometer (Shimadzu, Kyoto, Japan). A blank sample consisting of water and reagents was used as reference. Gallic acid (Sigma-Aldrich) (1.8; 2.2; 4.4; 6.6; 11.0; 16.4; 21.7 μg/mL and 2.6; 7.8; 10.5; 13.0; 26.0; 39.0; 64.8; 96.7; 128.2 μM) was used as standard to produce the calibration curves. The results were expressed in mg of gallic acid equivalents (GAE)/100 g of dry sample and μM of gallic acid equivalents.

### 2.3. Determination of Antioxidant Activity Using the 2,2-Diphenyl-1-picrylhydrazyl (DPPH) Radical Scavenging Method

The ability of the crude extract and Fraction 1 to scavenge DPPH (Sigma-Aldrich) free radicals was determined using the method described by Mensor [19]. Briefly, 29 μL of DPPH solution (0.3 mM in methanol) were mixed with the samples (710, 355, 178, 88.8, 44.4 and 22.2 μg·mL^−1^) in a 96-well microplate (100 μL). The reaction mixture was incubated for 30 min in the dark at room temperature. The absorbance was measured at 518 nm against a blank in an Elisa microplate reader (Bio-Rad Model 680 microplate reader). The free radical scavenging activity was determined by comparison with methanol and water controls. Quercetin (Sigma-Aldrich) was used as a reference compound. The percentage (%) of radical scavenging activity (RSA) was calculated using the following equation:

RSA = 100 − {[(*A_s_* + *A*_0_) × 100]/*A*_0_} (1)

where *A_s_* = absorbance of the sample and *A*_0_ = absorbance of the control. EC_50_ values denote the effective concentration of the sample required to scavenge 50% of DPPH free radicals, and it was graphically determined using a linear regression treatment. Captured DPPH was calculated using Lambert-Beer law with ε = 8317 M^−1^·cm^−1^ and ∆Abs (Abs_i_ − Abs_f_).

### 2.4. Iron Binding Ability

The *Fe II* chelating activityof the samples was determined with solutions of the crude extract, Fraction 1 (10 mg·mL^−1^), and Egb (*Ginkgo biloba* extract, 4 mg·mL^−1^) in methanol. The three mixtures were mixed with 1 mL of FeSO_4_·7H_2_O [Fe (II) 25 μM in PBS, pH = 7.0]. The mixture was incubated at room temperature for 10 minutes. Afterwards, the mixtures were scanned from 600 to 200 nm using a UV/VIS spectrophotometer (Shimadzu, Kyoto, Japan).

### 2.5. Tyrosinase Enzyme Inhibitory Activity

Reagents employed in the investigation of tyrosinase inhibitory activity were obtained from Sigma-Aldrich. First, the crude extract and Fraction 1 samples were dissolved in DMSO (1 mg·mL^−1^) for the following final concentrations in a total volume of 1.5 mL: 50.0, 16.0, 6.6 and 3.3 μg·mL^−1^, and L-DOPA (170 μmol·L^−1^), EDTA (22 μmol·L^−1^), tyrosinase (50–100 units) in PBS. The samples were then mixed with L-DOPA and EDTA in PBS solution, and when the tyrosinase solution was added to the mixture, the absorbance was immediately measured at 475 nm with a Shimadzu UV-VISspectrophotometer UV Mini 1240 (Kyoto, Japan). The reaction was monitored for 30 min, and 0.5 mmol·L^−1^ ascorbic acid was used as a positive control.

All experiments were performed in triplicate, and the obtained results were expressed as means ± SD. IC_50_ values were calculated from the equation generated by linear regression fit of the experimental data in Origin software (ANOVA statistical function).

## 3. Results and Discussion

The crude extract and Fraction 1 of *T. triangulare* were initially subjected to chemical screening [17]. The results indicated the presence of phenolic compounds, saponins and nitrogenous compounds.

### 3.1. Total Phenolic Contents

**Figure 1:**
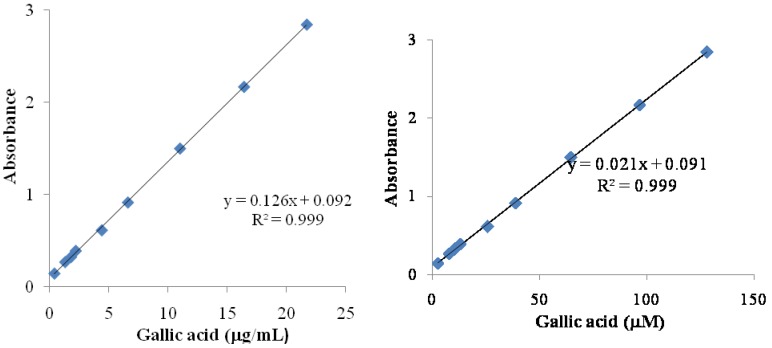
Standarts curves gallic acid in μg/mL and in μM. The equations used for express content phenolic was in μg/mL and in μM by gallic acid.

Folin-Ciocalteu phenol reagent reacts with phenols and a variety of other types of compounds include tertiary aliphatic amines, tryptophan, hydroxylamine, hydrazine, certain purines, and other miscellaneous organic and inorganic reducing agents [20]. The absorbance obtained for the crude extract was 0.128, and 0.250 for Fraction 1, these results indicated a higher concentration of phenols in Fraction 1, 585.3 mg/100 g dry sample, than that in the crude extract, 140.0 mg/100 g dry sample, as determined using a gallic acid standard curve (Figure 1). Because Fraction 1 was obtained after CHCl_3_ treatment, the concentration of polar compounds in it was higher compared to the crude extract, as expected.

### 3.2. DPPH Radical Scavenging Activity

**Figure 2:**
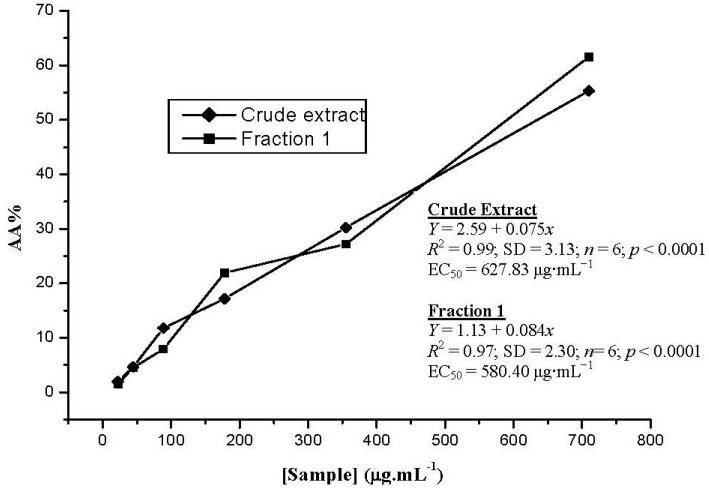
The comparative radical scavenging activity (RSA) of the crude extract and Fraction 1. The assays were performed with concentrations ranging from 710.0 to 22.2 μg·mL^−1^. Measurements were carried out in triplicate.

DPPH assays evaluate the ability of antioxidants to scavenge free radicals. The ability to donate a hydrogen atom is a primary characteristic of antioxidants. These antioxidants donate hydrogen atoms to free radicals, which converts the radicals into non-toxic species and therefore inhibits the propagation phase of lipid oxidation [21]. RSA of the crude extract and Fraction 1 were determined and found to have similar profiles (Figure 2). EC_50_ values for Fraction 1 and for the crude extract were 580.40 μg·mL^−1^ and 627.83 μg·mL^−1^, respectively.

**Figure 3:**
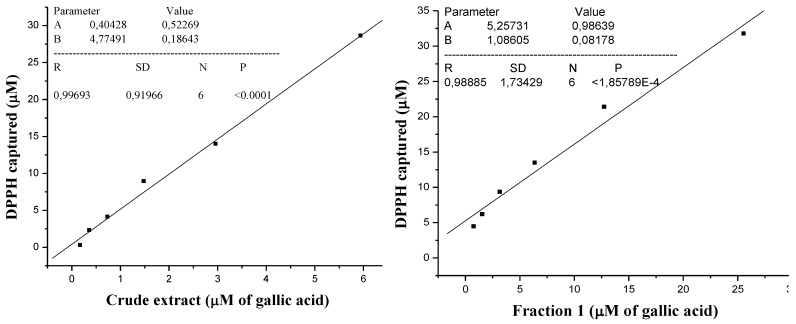
Amount DPPH captured *vs.* amount total phenolic for crude extract and *vs.* amount total phenolic for fraction 1 express in μM/gallic acid.

The gallic acid standard curve (Figure 1) was used for the determination of the total phenolic content for crude extract and Fraction 1; both of them were expressed in μM of gallic acid. The captured DPPH species was calculated (Table 1) and the values were used to obtain the captured DPPH *vs.* phenolic content graphics (Figure 3). The Pearson correlation coefficient (R), obtained between RSA and total phenolics, was 0.9969 for the crude extract and 0.9888 for Fraction 1, so RSA values were dependent on the total phenolic content. Thus, the DPPH assay results correlated with the total phenolic content, showing the importance of phenolic compounds as radical scavenger.

**Table 1:** The DPPH species captured and content phenolic express in μM/gallic acid. The absorbance initial was 0.429 for all test solution.

Crude Extract				Fraction 1			
[Sample] ^a^	Abs_f_	∆Abs ^b^	[DPPH_captured_] ^c^	[Sample] ^a^	Abs_f_	∆Abs ^b^	[DPPH_captured_] ^c^
0.18	0.427	0.002	0.24	0.80	0.392	0.036	4.40
0.37	0.410	0.019	2.26	1.60	0.378	0.050	6.13
0.74	0,395	0.034	4.08	3.20	0.351	0.077	9.3
1.50	0,355	0.074	8.90	6.41	0.317	0.111	13.42
2.97	0.313	0.116	13.94	12.79	0.251	0.177	21.32
5.95	0.191	0.238	28.60	25.59	0.165	0.264	31.70

^a^ (μM/gallic acid); ^b^ ∆Abs = Abs_i_ − Abs_f_; ^c^ μM.

### 3.3. Iron Binding Ability

**Figure 4:**
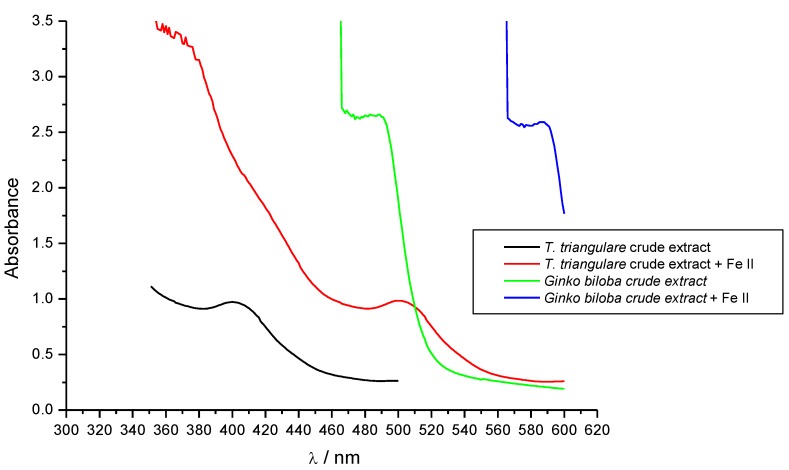
Binding ability of Fraction 1 from *T. triangulare* stem on Fe II.

*Ginkgo biloba* extract (Egb) is a phytotherapeutic agent used for the treatment of ischemic and neurological disorders. The redox properties of this extract are most likely due to the presence of flavonoids such as quercetin [22]. Thus, in this work, the UV-VIS spectroscopy was used to compare the iron binding ability of the crude extract to the *Ginkgo biloba* extract. The changes in the UV-VIS spectra (bathochromic effect) indicate that the crude extract is oxidized in a similar manner to the *Gingko biloba* extract (Figure 4). It suggests that the natural antioxidant phenolic compounds in the crude extract have a similar profile to those in Egb.

### 3.4. Tirosinase Inhibitory Activity

**Figure 5:**
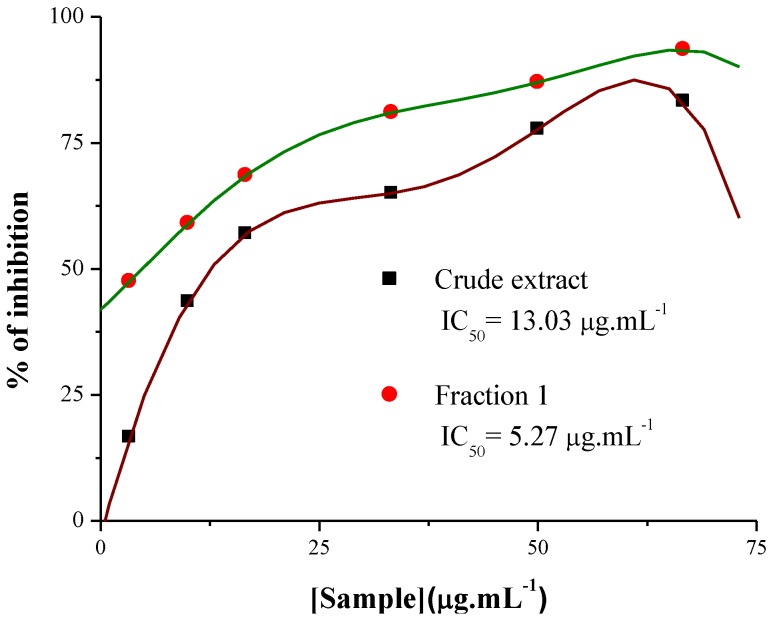
Tyrosinase inhibitory activity of the crude extract and Fraction 1 from *T. triangulare* stem.

**Figure 6:**
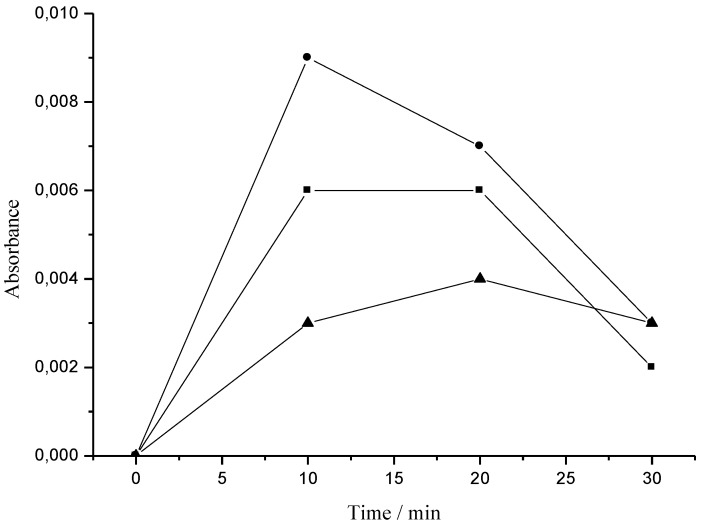
The inhibitory effect of ascorbic acid and Fraction 1 on tyrosinase. ▲: Fraction 1 (3.3 μg·mL^−1^) with L-DOPA and enzyme; ■: ascorbic acid (0.5 mmol·mL^−1^) with L-DOPA and enzyme; and ●: L-DOPA (0.17 mmol·L^−1^) with enzyme.

Tyrosinase is known as a key enzyme in melanin biosynthesis, which is involved in the determination of mammalian skin and hair color. In addition, tyrosinase causes the enzymatic browning of plant-derived foods, which decreases their nutritional quality and leads to economic losses [23]. In this investigation, the crude extract and Fraction 1 from *T. triangulare* stems were examined, and showed significant inhibition of L-DOPA oxidation according literature [24]. The ID_50_ values obtained were 13.3 μg·mL^−1^ for the crude extract, and 6.6 μg·mL^−1^ for Fraction 1 (Figure 5). Furthermore, Figure 6 shows that Fraction 1 is more effective at inhibiting tyrosinase than the positive control, ascorbic acid.

## 4. Conclusions

In summary, this study showed that *T. triangulare* stems contain phenolic compounds with high antioxidant power and represent a particularly promising source of bioactive antioxidants. Furthermore, the methanol/water extract of *T. triangulare* could be used as a natural antioxidant additive in food and pharmaceutical. The screening of the plant extracts, using DPPH free radical method, proved to be effective for the selection of those that could have an antioxidant activity. These extracts are rich in radical scavengers, known as antioxidants, so studies are needed to identify which special metabolites are responsible for the antioxidant activity of the species. In addition, studies such as lipid peroxidation and *in vivo* assays, are essential to characterize them as biological antioxidants.
